# Some Military Hospital Gazettes

**Published:** 1916-10-21

**Authors:** 


### Some Military Hospital Gazettes.

THE "ANZAC BULLETIN."

This is a hospital gazette which is not a hospital gazette. The object of the four-page newspaper is to provide Australians who are on active service with a brief summary of up-to-date Commonwealth news, and with any other information which is likely to interest them; it is distributed gratuitously to members of the Force in France and England, and in this way it arrives every Monday, Wednesday, and Friday at British hospitals where there are wounded Australians. The columns of the issue before us are largely taken up by news of the Referendum Bill, and an excellent account of the fighting with which the Australians were so gloriously associated at Mouquet Farm on the same day that the British took Guillemont. This description is from the pen of Mr. C. E. W. Bean, the accredited correspondent with the Australian Forces. It contains a splendid story, which no one can read and remain unmoved, of how a trench was won through a sergeant borrowing the well-known walking-stick of the badly wounded senior captain (the officer's only weapon), and with it waving the boys on to victory. The *Bulletin*, with its fresh news of home, must be a tonic to many an Australian patient in our hospitals.

"CRAIGLEITH HOSPITAL CHRONICLE."

A great writer receives his crowning tribute when he attracts the attention of the able caricaturist and becomes a subject for parody, since parody and caricature are only understandable of men and styles familiar to the mass of a nation, and no man can become so famous as to be familiar in this personal way without marked ability. Thus not only readers of *Land and Water* but Mr. Hilaire Belloc himself are certain to enjoy a really stirring parody of him entitled "The Penultimate Phase: being a review of the second year of war, after the later style of Mr. Hilaire Belloc," by Bertram Smith, in the October number of the *Craigleith Hospital Chronicle*. It is admirable fooling, sufficiently imitative of Mr. Belloc's style to make hilarious reading, and sufficiently exaggerating his mannerisms, parentheses, italics, and the alternation of long sentences with sentences of three or four words only, to be, like all good parody, a criticism as well as a joke. This is only one good thing among many in a gazette, as we have often had occasion to remark, that is at once the most ambitious and best written of its class.

THE "SOUTHERN CROSS."

The monthly journal of the 1st Southern General Hospital, Birmingham, known as the *Southern Cross*, fills its October number with sketches and "happenings," although we are glad to see that Pepys still persists behind the scenes, and that material is not yet exhausted for the series of "Who's Zoo" cartoons. The illustrations are composed of drawings and photographs, and among the latter is one depicting the presentation of the D.C.M. to Sergt. A. McKay, 4th Yorks, at Hill Crest, Radford Road, Coventry. There is the second of a new series of black-and-white drawings amusingly entitled "Muddley Road" celebrities. In fact, one of the most pleasant results of the war hospital gazettes has been a revival of black-and-white illustrations, which are in every sense an improvement on the products of the commercial camera. We hope that W. E. R. (?), whose signature to the sketch of No. 2 of the Muddley Road celebrities is imperfectly readable, will continue this class of work, and that his success may encourage its development on a larger scale in the *Southern Cross*. Inset illustrations and thumbnail sketches always make a page attractive, and nothing enlivens a gazette so much as a number of small drawings. They are the modern equivalent of the old decorative designs, the tradition of which Mr. Charles Ricketts has put to such charming use in many modern books.

